# Neurological Complications in Children with Moyamoya Disease—Case Report and Literature Review

**DOI:** 10.3390/jcm15062242

**Published:** 2026-03-16

**Authors:** Ioana Grigore, Lăcrămioara Ionela Butnariu, Thomas Gabriel Schreiner, Vasile Valeriu Lupu, Ancuta Lupu, Ludmila Darie, Elena Țarcă, Alexandra Vătămănelu, Raul Andrei Crețu, Ecaterina Grigore

**Affiliations:** 1 Neurology, “St. Mary” Children Emergency Hospital, 700309 Iasi, Romania; ioanag74@yahoo.com (I.G.); darie.ludmila@gmail.com (L.D.); 2 Grigore T. Popa University of Medicine and Pharmacy, 700115 Iasi, Romania; lacrybutnariu@gmail.com (L.I.B.); valeriulupu@yahoo.com (V.V.L.); anca_ign@yahoo.com (A.L.); elena.tuluc@umfiasi.ro (E.Ț.); vatamanelu.alexandra@gmail.com (A.V.); egrigore2002@gmail.com (E.G.); 3 Regional Center for Medical Genetics Iasi, Saint Mary’s Emergency Children Hospital, 700309 Iasi, Romania; 4 First Neurology Clinic, “N. Oblu” Clinical Emergency Hospital, 700309 Iasi, Romania; 5 Department of Surgery II—Pediatric Surgery, Saint Mary’s Emergency Children Hospital, 700309 Iasi, Romania

**Keywords:** moyamoya disease, pediatric stroke, epilepsy, headache, cognitive impairment, psychiatric manifestations

## Abstract

**Background**: Moyamoya disease (MMD) is a rare, progressive cerebrovascular arteriopathy characterized by stenosis and occlusion of the distal internal carotid arteries with the development of compensatory collateral networks. In children, MMD is a major cause of ischemic stroke; however, neurological morbidity frequently extends beyond cerebrovascular events to include epilepsy, headache, cognitive impairment, and psychiatric manifestations. Data regarding the long-term evolution of these complications in Caucasian pediatric patients remains limited. **Case Report**: We present the longitudinal case of a Caucasian female diagnosed with advanced MMD after an ischemic stroke at the age of 7 years, followed by indirect surgical revascularization (encephalo-duro-arterio-synangiosis) and chronic antiplatelet therapy. Four years later, she developed recurrent focal aware sensory–motor seizures associated with chronic post-ischemic cortical injury. Despite stable vascular imaging and absence of recurrent infarction, the patient experienced persistent neurological sequelae, including residual spastic hemiparesis, episodic tension-type headaches, and evolving neuropsychological complications. Cognitive assessment initially suggested mild neurocognitive impairment, with subsequent improvement during adolescence. In late follow-up, prominent anxiety symptoms emerged, and psychiatric evaluation confirmed panic disorder requiring psychological and pharmacological support. The patient remained neurologically stable into adulthood under continued multidisciplinary care. This case illustrates the broad spectrum of neurological and psychiatric complications that may accompany pediatric MMD, even in the absence of new ischemic events. The accompanying literature review emphasizes that epilepsy, headache, cognitive dysfunction, and psychiatric disorders represent clinically significant components of the long-term disease burden in children with MMD. **Conclusions**: Pediatric moyamoya disease should be regarded not only as a cause of childhood stroke, but also as a chronic condition with long-term epileptic, cognitive, and psychiatric sequelae that may evolve independently of recurrent ischemic injury. By providing longitudinal follow-up from childhood into adulthood in a Caucasian patient, this report underscores the importance of integrating neuropsychological and psychiatric surveillance into standard care pathways, alongside vascular and surgical management, to better address the full spectrum of morbidity and improve quality of life.

## 1. Introduction

Moyamoya disease (MMD) was first described in the Japanese medical literature by Kudo in 1957. The term moyamoya, meaning “a puff of smoke,” was later introduced by Suzuki and Takaku in 1969 to describe the characteristic angiographic appearance of abnormal collateral vessels. Since then, clinical awareness and understanding of this rare cerebrovascular disorder have increased substantially [1].

Moyamoya disease is a chronic, progressive vasculopathy characterized by idiopathic stenosis or occlusion of the terminal portion of the internal carotid arteries and their proximal branches within the circle of Willis. This arterial narrowing leads to the development of fragile compensatory collateral networks, which are prone to both cerebral hypoperfusion and hemorrhage [2]. Consequently, patients with MMD are at increased risk of ischemic stroke due to impaired cerebral blood flow, as well as intracranial hemorrhage resulting from rupture of the abnormal collateral vessels [3,4,5].

When similar vascular changes occur in association with an underlying systemic or acquired condition, the disorder is classified as moyamoya syndrome (MMS) rather than idiopathic disease. Moyamoya syndrome has been reported in association with genetic disorders (e.g., sickle cell disease, neurofibromatosis type 1, and Down syndrome), infectious conditions (e.g., congenital or postnatal cytomegalovirus infection and tuberculosis), renal and renovascular abnormalities, congenital heart disease, endocrine disorders, and prior cranial irradiation. Distinguishing between MMD and MMS is clinically relevant, as associated comorbidities may influence both management and prognosis [6,7].

The incidence of MMD remains relatively low in Caucasian populations, with an estimated annual incidence of approximately 0.1 cases per 100,000 individuals in both pediatric and adult groups [8]. Despite its rarity, MMD represents an important cause of pediatric cerebrovascular disease and has been implicated in approximately 6–10% of childhood ischemic strokes and transient ischemic attacks (TIAs), particularly in cases with a high risk of recurrence.

Given the low prevalence of moyamoya vasculopathy in non-Asian populations, diagnosis may be delayed, and available data regarding clinical presentation, long-term progression, and neurological outcomes in affected children remain limited. Neurological complications extend beyond ischemic events and may include epilepsy, headache, cognitive impairment, and psychiatric manifestations, all of which can significantly affect quality of life and functional prognosis [9,10,11].

The present study reports a pediatric case of moyamoya disease with long-term follow-up into adolescence and early adulthood, illustrating a complex neurological and neuropsychiatric course. In addition, we provide a narrative review of the literature focusing on the main neurological complications of moyamoya disease in children, emphasizing the importance of early recognition and multidisciplinary management to reduce long-term morbidity.

## 2. Case Report

A female patient, currently aged 17 years and 11 months, was first evaluated in our Neurology Department of “Sf. Maria Emergency Clinical Hospital for Children”, Iași, Romania, at the age of 11 years due to new-onset recurrent focal aware sensory–motor seizures, classified according to the International League Against Epilepsy (ILAE) 2017 [12] framework (Table 1). There was no history of parental consanguinity, and the family history was negative for cerebrovascular or neurological disorders.

**Figure 1:**
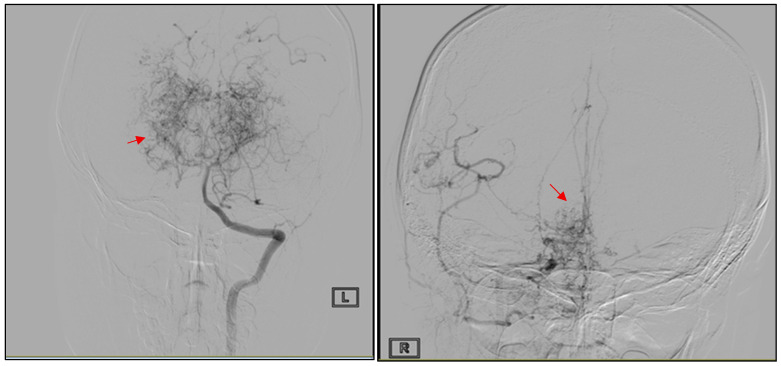
Carotid arteriography at the age of 7 demonstrating advanced moyamoya vasculopathy, characterized by bilateral occlusion of the terminal internal carotid arteries with prominent basal collateral vessel formation (“moyamoya” vessels, red arrows).

At stroke onset, motor examination documented severe left hemiparesis, with muscle strength graded 2/5. The neurological deficit was predominantly motor, without cranial nerve or language involvement. According to the available clinical documentation, the neurological impairment corresponded to a National Institutes of Health Stroke Scale (NIHSS) score exceeding 10 at admission, reflecting significant motor deficit. At the time of discharge, after partial neurological recovery, the deficit corresponded to an estimated NIHSS score of approximately 6, driven mainly by left upper and lower limb weakness. Neuroimaging performed at that time confirmed a right parieto-occipital arterial ischemic stroke, and cerebral angiography established the diagnosis of MMD (Figure 1). The angiographic findings were consistent with Suzuki stage IV moyamoya disease, demonstrating advanced steno-occlusive changes in the terminal internal carotid arteries with extensive collateral vessel formation. Additional collateral circulation arose from the ophthalmic arteries and lenticulostriate perforators, partially revascularizing the anterior cerebral artery territories, with further collateral supply involving distal posterior cerebral artery branches. Following this diagnosis, the patient underwent indirect surgical revascularization with encephaloduroarteriosynangiosis (EDAS). Long-term antiplatelet therapy with acetylsalicylic acid (75 mg daily) was initiated following neurosurgical evaluation after the ischemic event and maintained as part of the long-term secondary stroke prevention strategy in moyamoya disease.

Over the following four years, the patient participated in regular physical rehabilitation and gradually improved. On examination at age 11, neurological evaluation revealed a minor left hemiparesis, with muscle strength graded 4/5 on the left side and 5/5 on the right according to the MRC, associated with subtle spastic hypertonia. Deep tendon reflexes were present bilaterally, with pyramidal signs on the left, including a positive Babinski response, as well as Chaddock and Oppenheim signs. Cranial nerve examination was normal, and no sensory impairment was detected. The patient was able to ambulate independently over moderate distances without assistive support. Coordination was largely preserved, with only minor hypometria noted during finger-to-nose testing. No extrapyramidal manifestations were observed.

Neuropsychological evaluation performed at the age of 11, including the WISC-V and the ROCF, suggested mild-to-moderate neurocognitive impairment, consistent with subtle cognitive difficulties following early childhood ischemic brain injury within the DSM-5 framework for neurocognitive disorders. The Full Scale IQ on the WISC-V was 78, corresponding to borderline intellectual functioning. Performance on the Rey–Osterrieth Complex Figure Test indicated mild deficits in visuospatial organization and visual memory, consistent with the structural right hemispheric lesion.

A comprehensive etiological work-up was conducted to exclude alternative causes of pediatric stroke and secondary moyamoya syndrome. Screening for disorders of lipid and cholesterol metabolism was normal. Coagulation studies, including antithrombin III, homocysteine levels, prothrombin time, activated partial thromboplastin time, thrombin time, and factor V Leiden mutation testing, were within normal limits. Antiphospholipid antibodies and lupus anticoagulant were absent. Testing for the prothrombin gene (G20210A) mutation was also negative. Additional inherited thrombophilia investigations, such as protein C and protein S activity, were negative. Furthermore, there was no clinical or laboratory evidence suggestive of systemic vasculitis or inflammatory arteriopathy. Genetic testing for moyamoya-associated variants, including RNF213, was not accessible in our center during the diagnostic work-up. In addition, no underlying systemic disorder commonly associated with moyamoya syndrome was identified. The patient had no clinical features suggestive of neurofibromatosis type 1 or Down syndrome, no history of cranial irradiation, and no hematological evidence of sickle cell disease. Thyroid function was normal, and there were no signs of chronic infection or systemic inflammatory disease. Overall, these findings supported the diagnosis of idiopathic moyamoya disease rather than moyamoya syndrome.

**Figure 2:**
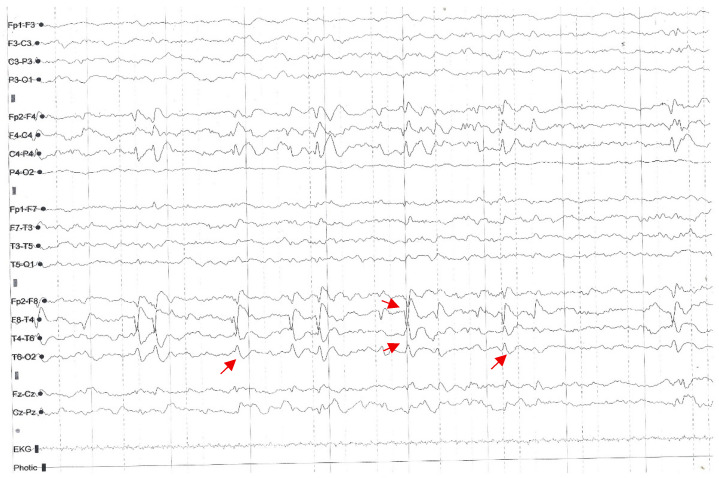
Sleep EEG showing biphasic spike discharges in the right fronto-central–parietal (F-C-P) and right fronto-temporal (F-T) leads (red arrows).

Electroencephalography (EEG) during wakefulness was normal. However, sleep EEG demonstrated biphasic spike discharges in the right fronto-central–parietal (F-C-P) and right fronto-temporal (F-T) leads (Figure 2).

**Figure 3:**
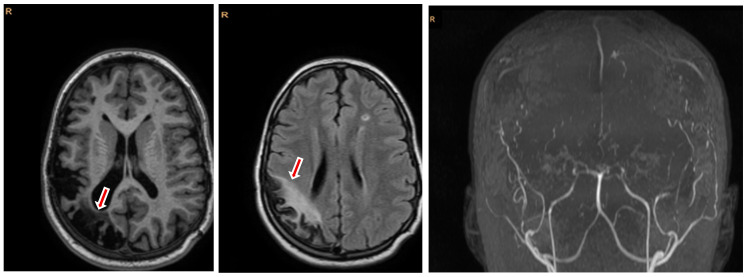
Brain MRI (axial T2-FLAIR sequences, (**left**) and (**middle**)) and time-of-flight MRI angiography (**right**) demonstrating chronic right parieto-occipital ischemic sequelae (red arrows) and vascular changes consistent with moyamoya disease: FLAIR, fluid-attenuated inversion recovery; MR, magnetic resonance imaging; R, right.

Brain magnetic resonance imaging (MRI) at the age of 11 revealed chronic post-ischemic sequelae, including a large right parieto-occipital cortico-subcortical porencephalic cavity with adjacent gliosis and mild secondary dilation of the right occipital horn. Additional small ischemic sequelae lesions were observed in the frontal subcortical white matter on FLAIR sequences. Three-dimensional time-of-flight MR angiography confirmed features characteristic of moyamoya vasculopathy, with attenuation of the anterior circulation branches and collateral vessel development, while venous sinuses appeared normal (Figure 3).

Treatment was initiated with topiramate, gradually titrated to a dose of 100 mg/day, given the presence of focal seizures in the setting of a structural post-ischemic lesion and the concomitant complaint of headache. Although potential cognitive adverse effects were considered, the medication was introduced with slow dose escalation and close clinical monitoring. Serial neuropsychological evaluations did not demonstrate any clinically meaningful treatment-related cognitive deterioration; rather, cognitive performance improved over the course of follow-up. Because seizure recurrence persisted during follow-up, clonazepam (2 mg/day) was introduced as adjunctive therapy after three months. At that stage, seizure frequency was estimated at approximately two focal sensory–motor episodes per month. In parallel, physical rehabilitation was recommended and continued for the residual left spastic hemiparesis. In parallel, physical rehabilitation was recommended and continued for the residual left spastic hemiparesis.

At the age of 15 years and 11 months, the patient underwent neurological re-evaluation because of recurrent episodes of tension-type headache, sleep disturbances, and persistent focal seizures despite ongoing antiseizure therapy. Repeat brain MRI and MR angiography demonstrated no significant interval changes compared with previous examinations, with no evidence of new ischemic lesions or hemorrhagic events. During hospitalization, transient elevations in blood pressure were noted. These values decreased after symptomatic treatment with a single dose of furosemide, and no long-term antihypertensive therapy was required. Cardiac assessment revealed minimal mitral regurgitation and grade I tricuspid regurgitation. In addition, abdominal computed tomography angiography showed no abnormalities of the abdominal aorta, renal arteries, or mesenteric vessels, thereby excluding renovascular causes of hypertension.

Psychological reassessment at that time indicated normal cognitive development, although emotional dysregulation and anxiety-related symptoms were reported.

Given the persistence of seizure activity, antiseizure medication was adjusted. Clonazepam was gradually tapered, and levetiracetam was introduced and titrated to a final dose of 1000 mg/day. Furthermore, due to ongoing headaches and incomplete seizure control, the dose of topiramate was progressively increased to 200 mg/day. Treatment with acetylsalicylic acid was continued, and psychological counseling was recommended.

At the age of 17 years and 11 months (evaluation performed in 2024), the patient’s seizures were well controlled under the current regimen, allowing gradual reduction in levetiracetam. Follow-up brain MRI and MR angiography again demonstrated stable chronic post-ischemic sequelae, without new cerebrovascular events.

In late adolescence, the patient developed prominent anxiety symptoms, and psychiatric consultation resulted in a diagnosis of panic disorder according to DSM-5 clinical criteria. No standardized anxiety rating scales were administered during that evaluation. Initial management included psychological counseling and treatment with a selective serotonin reuptake inhibitor (sertraline); however, clinical response was limited, with persistent acute panic episodes. Therefore, short-term treatment with alprazolam was initiated under psychiatric supervision to provide more rapid symptomatic control. Benzodiazepine therapy was used cautiously and as adjunctive treatment, given its known role in the short-term management of severe anxiety symptoms.

After reaching the age of 18 years, the patient was transitioned to adult neurology care and has remained under supervision; no significant clinical deterioration or new neurological events have been reported during the subsequent two-year follow-up period.

## 3. Discussion

In children, the clinical spectrum of MMD is predominantly shaped by cerebral ischemic events, including ischemic stroke and TIAs, whereas hemorrhagic presentations are less frequent in this age group. Beyond vascular events, a wide range of neurological manifestations may occur, such as epileptic seizures, headache, cognitive impairment, and movement disorders [22,23]. Importantly, the relative frequency of these complications appears to be age-dependent [5], and initial presentation may vary considerably, ranging from focal neurological deficits due to stroke or TIAs to seizures or headache [7].

Gatti et al. reported that among pediatric patients with MMD or MMS, ischemic stroke or TIAs represented the first clinical manifestation in 55% of cases, while seizures and headache accounted for 14% and 12%, respectively [7]. Notably, more than one quarter of children presented with multiple symptoms at diagnosis, most commonly a combination of cerebrovascular events with headache or epilepsy, highlighting the heterogeneous clinical onset of the disease [7].

Clinical manifestations related to ischemic stroke or TIAs are typically the earliest indicators of MMD [24,25] and may be precipitated by hyperventilation-related triggers such as crying, respiratory infections, fatigue, or emotional stress [26]. Common presenting deficits include focal motor weakness, dysarthria, and aphasia [23,27]. In a cohort of 22 pediatric patients, Boulter et al. observed that stroke was the initial presentation in 63.6% of cases, whereas TIAs accounted for 18.2% [24]. Similarly, Baba et al. reported that nearly 80% of children younger than 10 years presented with ischemic symptoms [28]. In the present case, the disease first manifested at the age of 7 years with sudden-onset left hemiparesis secondary to an ischemic stroke.

Epileptic seizures represent an additional and clinically relevant complication in pediatric MMD, although their mechanisms remain multifactorial. Seizures may occur as an early manifestation related to ischemic injury within the internal carotid artery territory [29]. Furthermore, a close association between stroke and epilepsy has been emphasized, with many cases meeting criteria for post-stroke epilepsy when seizures develop after cerebral infarction [1]. Seizures may also emerge following revascularization surgery, potentially reflecting altered cortical excitability during postoperative hemodynamic reorganization [29].

Despite their clinical relevance, the reported incidence of epilepsy in MMD varies substantially across studies, reflecting heterogeneity in patient populations, age distribution, and follow-up duration. Published rates range from as low as 0.9% to over 40% in selected cohorts [30,31]. In pediatric populations, seizure prevalence has been estimated between 11% and 18% [1,32,33,34,35], whereas adult series report slightly higher rates of 18–24% [33,34]. Boulter et al. found that seizures were the presenting symptom in 18.2% of children with MMD or MMS [24], while Ma et al. documented a preoperative seizure incidence of 18.1% in pediatric patients undergoing surgical treatment [32].

Long-term follow-up data further suggest that epilepsy may develop during the disease course. Gatti et al. observed seizures in 40% of children after a mean follow-up of 6.9 years, with onset occurring either at diagnosis, secondary to stroke, or months to years later during progression [7]. Similarly, Po et al. reported seizures in 15% of pediatric patients, with several cases emerging after diagnosis or following surgical intervention [11].

Focal seizures appear to be the most common semiology in moyamoya-associated epilepsy, accounting for approximately three quarters of cases in both Nakase’s and Bautista-Lacambra’s series [31,35]. Less frequently, generalized epilepsy syndromes, including absence seizures or Lennox–Gastaut syndrome, have been described (Table 2). In our patient, the first epileptic seizure occurred at the age of 11 years, approximately four years after the initial diagnosis of MMD. Seizures were focal with sensory–motor involvement of the left side of the body and were ultimately controlled with antiseizure medication.

In most pediatric patients with MMD, epilepsy can be adequately managed with antiseizure medication. Gatti et al. reported that among children with MMD and associated seizures, 94% received pharmacological treatment, and 88% achieved seizure freedom [7]. Nevertheless, certain clinical factors have been linked to a less favorable epilepsy prognosis, particularly very early seizure onset (often before 3 years of age) and the presence of cortical structural lesions [42]. Moreover, although uncommon, drug-resistant epilepsy has been described in up to 30% of cases, especially when seizures arise from an established ischemic substrate [31,43].

Headache represents another frequent and often underrecognized manifestation in pediatric MMD, likely reflecting impaired cerebrovascular reserve and chronic cerebral hypoperfusion [44]. Reported prevalence ranges from 20% to 22% among affected children [45,46]. Clinically, headache may resemble either tension-type headache or migraine-like attacks [19]. In a cohort study of children with MMD or MMS, Po et al. identified headache in 25 of 61 patients, including cases of migraine, tension-type headache, mixed headache phenotypes, and nonspecific presentations [11].

Despite its clinical relevance, there remains no consensus regarding optimal symptomatic management. Kraemer suggested that beta-blockers and calcium channel blockers should be used with caution, given the potential risk of systemic hypotension and secondary worsening of cerebral perfusion [47]. Conversely, Aihara proposed that aspirin, which is commonly administered as long-term antiplatelet therapy in MMD, may exert a beneficial effect on headache episodes through inhibition of platelet activation [48]. In addition, Canavero et al. highlighted that certain antiseizure medications, such as topiramate, lamotrigine, or sodium valproate, may be particularly useful in patients presenting with both epilepsy and migraine-like headaches, as these agents provide dual therapeutic benefit [19].

In the present case, the patient experienced intermittent headache episodes during follow-up, which contributed to escalation of topiramate dosage and the recommendation for continued psychological support. Notably, she also remained on chronic acetylsalicylic acid therapy throughout adolescence.

Psychiatric and neuropsychological complications have increasingly been recognized as an important component of the clinical spectrum of MMD. Psychiatric symptoms, including cognitive impairment, depressive features, and anxiety disorders, have been reported in approximately 20–60% of patients [49]. In some cases, psychiatric manifestations may even represent an early presenting feature, as summarized in Table 3.

Cognitive impairment represents one of the most prevalent and clinically impactful non-vascular complications of moyamoya disease in childhood [16,54]. Neurocognitive deficits have been reported in approximately 30–70% of pediatric cases [55,56], with younger age at disease onset consistently associated with poorer long-term outcomes [57,58]. In children with MMD, irreversible ischemic brain injury may adversely affect multiple cognitive domains, including global intellectual functioning, memory, processing speed, and visuospatial abilities [55,56,57,58,59]. Bowen et al. further suggested that the severity of cognitive dysfunction is closely related to the degree of cerebral hypoperfusion, supporting the concept that chronic hemodynamic compromise contributes substantially to neurodevelopmental vulnerability [57].

Importantly, surgical revascularization, either through direct or indirect bypass procedures, has been shown to improve cerebral hemodynamics and reduce the risk of recurrent ischemic events, with several reports indicating stabilization or partial improvement of cognitive performance following intervention [60,61]. Improvements have been described across different domains, including language function [55], verbal memory [62], and frontal lobe-mediated executive abilities such as attention and cognitive flexibility (Table 4) [63]. Nevertheless, outcomes remain heterogeneous, and the extent of recovery likely depends on factors such as timing of surgery, baseline cerebral perfusion, and the presence of established cortical infarction.

In the present case, a neuropsychological assessment performed at the age of 11 suggested mild neurocognitive impairment, likely reflecting the consequences of early childhood ischemic brain injury. Notably, cognitive functioning improved over time, and subsequent evaluation at the age of 15 years and 11 months indicated age-appropriate cognitive development. During adolescence, the patient developed additional psychiatric manifestations, including emotional dysregulation and, later, panic disorder, which required psychological counseling and psychiatric management. This clinical course illustrates the evolving neuropsychological burden of pediatric moyamoya disease (MMD), extending beyond purely vascular or motor sequelae.

Movement disorders have also been described as part of the pediatric moyamoya spectrum and may, in some instances, precede diagnosis [11]. Koruda et al. emphasized involuntary movements as a relevant, although relatively uncommon, manifestation in children with MMD [2]. Similarly, Borah et al. reported a pediatric case presenting with choreiform movements in addition to hemiparesis, highlighting the diversity of neurological phenotypes at disease onset [23]. Furthermore, Gatti et al., in a cohort of 65 children with MMD or moyamoya syndrome (MMS), identified movement disorders in a small subset of patients, including dyskinesia, dystonia, and myoclonus [7]. In contrast, no movement abnormalities were observed in our patient, either at presentation or during longitudinal follow-up.

In addition to ischemic and functional neurological manifestations, vascular complications such as intracranial aneurysm formation have also been reported in association with moyamoya disease. Pediatric series have described aneurysms arising in fragile collateral networks or in major intracranial arteries subjected to altered hemodynamic stress. Liu et al. reported that intracranial aneurysms may occur in children with MMD and represent an additional source of hemorrhagic risk, particularly when associated with abnormal collateral circulation [65]. Although no aneurysmal lesions were identified in the present patient, this potential complication further highlights the complexity of the vascular pathology and the importance of careful long-term neurovascular monitoring.

A limitation of this case report is the absence of RNF213 mutation analysis, as genetic testing for moyamoya-associated variants was not available at our center during the diagnostic evaluation. Another limitation is the absence of extended psychiatric follow-up after the patient transitioned to adult care at the age of 18 years, which limited further assessment of the progression and underlying mechanisms of the reported panic disorder.

## 4. Conclusions

Moyamoya disease represents an important cause of pediatric cerebrovascular pathology and is associated with substantial long-term neurological and neuropsychological morbidity. As illustrated by the present case and supported by the existing literature, the clinical manifestations of the disease frequently extend beyond ischemic motor deficits and may include epilepsy, headache, cognitive impairment, and psychiatric complications developing over time. The longitudinal evolution observed in this patient highlights the complex and dynamic spectrum of neurological and neuropsychiatric manifestations that may occur throughout childhood and adolescence in moyamoya disease. This report provides a detailed clinical illustration of how multiple complications including post-stroke epilepsy, evolving cognitive profile, and later psychiatric manifestations may develop sequentially in a single pediatric patient. Such observations emphasize the importance of early recognition, appropriate revascularization strategies, and multidisciplinary long-term follow-up to reduce neurological sequelae and support optimal neurodevelopmental outcomes.

Further research, particularly multicenter prospective studies, is needed to better characterize the natural history of pediatric moyamoya disease, clarify genotype–phenotype correlations, and identify predictors of long-term neurological and psychiatric outcomes. In addition, the development of standardized approaches to medical, surgical, and neuropsychological management may contribute to more consistent care and improved quality of life for affected children.

## Figures and Tables

**Table 1:** Clinical Scales, Classifications, and Diagnostic Investigations Used.

Category	Tool/Investigation	Purpose in This Case	Result/Interpretation
Moyamoya severity [13,14]	Suzuki angiographic staging system	Classification of angiographic progression of Moyamoya disease (MMD)	Advanced disease consistent with Suzuki stage IV
Seizure semiology [12,15]	ILAE 2017 [12] seizure classification	Standardized classification of seizure type	Focal aware sensory–motor seizures
Motor deficit assessment [16]	Medical Research Council (MRC) scale	Quantification of muscle strength over time	Left hemiparesis with MRC = 4/5
Stroke severity [17]	National Institutes of Health Stroke Scale (NIHSS)	Estimation of neurological deficit severity	Predominantly motor deficit, estimated NIHSS ≈ 6
Cognitive assessment [18,19]	Wechsler Intelligence Scale for Children (WISC-V)	Evaluation of intellectual functioning in childhood	Mild neurocognitive impairment at age 11 WISC-V-78
Visuospatial/memory testing [18,20]	Rey–Osterrieth Complex Figure Test (ROCF)	Assessment of visuospatial construction and recall	Subtle deficits compatible with post-ischemic sequelae
Psychiatric framework [18]	DSM-5 diagnostic criteria	Diagnosis of panic disorder in late adolescence	Panic disorder diagnosed at age 17
Thrombophilia screening	Factor V Leiden mutation	Exclusion of inherited thrombophilia	Negative
	Prothrombin gene mutation (G20210A)	Exclusion of inherited thrombophilia	Negative
	Antithrombin III, homocysteine	Hypercoagulability evaluation	Hypercoagulability evaluation
Autoimmune screening	Antiphospholipid antibodies, lupus anticoagulant	Exclusion of antiphospholipid syndrome	Absent
Additional thrombophilia tests	Protein C/Protein S activity	Further thrombophilia evaluation	Negative
Vasculitis exclusion	Clinical and laboratory assessment (ESR/CRP not suggestive)	Exclusion of inflammatory arteriopathy	No evidence of systemic vasculitis
Moyamoya syndrome exclusion	Clinical evaluation for neurofibromatosis type 1 (NF1)/Down syndrome	Exclusion of syndromic moyamoya	No clinical features present
	History of cranial irradiation	Exclusion of secondary moyamoya	Absent
	Hematological evidence of sickle cell disease	Exclusion of sickle cell–associated moyamoya	Not suggestive clinically
	Thyroid/endocrine screening	Exclusion of endocrine-associated MMS	Normal thyroid function
Genetic testing [21]	Ring Finger Protein 213 (RNF213) mutation analysis	Moyamoya-associated variant screening	Not performed

**Table 2:** Pediatric cases of MMD and epileptic seizures in the literature data: neurofibromatosis type 1, NF I; generalised tonic–clonic seizures, GTCS; y, years; F, female; M, male.

Authors	Child Age, Sex	Onset Age of Seizures	Type of Seizures	Treatment	Associated Causes
Borah et al. [23]	9 y, F	9 y	Focal seizures	Levetiracetam	
Kaushik et al. [36]	6 y, M	3 y	Nocturnal tonic seizure, tonic drop attacks, and head drops	Sodium valproate, Levetiracetam, Lamotrigine, Clonazepam	
Kikuta et al. [37]	6 y, F	4 y	Absence seizures	Sodium valproate	
Zhao et al. [38]	2 y, M	2 y	Focal seizures	Levetiracetam	NF I Hydrocephalus
Talbot et al. [39]	16 y, M	16 y	GTCS	Sodium valproate, Levetiracetam	CLN6 biallelic mutations
Shetty-Alva et al. [40]	7 y, M 5 y, M	7 y 5 y	Focal seizures Focal seizures	Carbamazepine Phenobarbitol	
Mohapatra et al. [41]	14 y, M	9 y	Focal seizures	Oxcarbazepine	

**Table 3:** Literature review regarding the onset of psychiatric manifestations in children with MMD: y, years; M, male.

Authors	Age, Sex	Onset of Psychiatric Manifestations
Klasen et al. [50]	12 y, M	Episodes of acute transient psychosis triggered by physical exertion
Dn et al. [51]	11 y, M	Sudden onset of aggression and abnormal behaviors
Behere et al. [52]	16 y, M	Mania without psychotic symptoms
Gnanavel [53]	8 y, M	Psychosis symptoms (irritability, frequent anger outbursts, suspiciousness, muttering to self, hearing voices of cartoon characters)
Mohapatra [41]	14 y, M	Hyperactivity, easy distractibility, inattention, irritability, emotional lability

**Table 4:** Literature review regarding cognitive impairment in children with MMD.

Authors	Number of Patients	Domains of Cognition Tested and Improved
Deckers et al. [55]	21	Attention, executive and visuospatial function, processing speed, intelligence, language, memory, and working memory
Hsu et al. [62]	23	Intelligence, verbal comprehension, memory, working memory, verbal learning, processing speed, executive function, perceptual organization
Kin et al. [63]	55	Intelligence, visuospatial and executive function, attention, and memory
Lee et al. [64]	65	Intelligence and visual memory

## Data Availability

The data presented in this study are not publicly available due to privacy and ethical restrictions related to patient confidentiality.

## Abbreviations

3D-TOF MRA	Three-dimensional time-of-flight MR angiography
ACA	Anterior cerebral artery
AIS	Arterial ischemic stroke
DSM-5	Diagnostic and Statistical Manual of Mental Disorders, Fifth Edition
EDAS	Encephaloduroarteriosynangiosis
EEG	Electroencephalogram
FLAIR	Fluid-attenuated inversion recovery
ILAE	International League Against Epilepsy
L	Left
MMD	Moyamoya disease
MMS	Moyamoya syndrome
MRA	Magnetic resonance angiography
MRC	Medical Research Council scale
MRI	Magnetic resonance imaging
NIHSS	National Institutes of Health Stroke Scale
PCA	Posterior cerebral artery
R	Right
TIA	Transient ischemic attack
